# Editorial: Rhizosphere interactions on soil carbon cycle under stress environments

**DOI:** 10.3389/fpls.2024.1466501

**Published:** 2024-07-26

**Authors:** Junjie Lin, Zhichao Xia, Peng Wang, Jiayu Lu, Chuntao Yin

**Affiliations:** ^1^ School of Environment and Natural Resources, Zhejiang University of Science and Technology, Hangzhou, Zhejiang, China; ^2^ Anhui Provincial Key Laboratory of Forest Resources and Silviculture, School of Forestry & Landscape Architecture, Anhui Agricultural University, Hefei, China; ^3^ Huitong Experimental Station of Forest Ecology, CAS Key Laboratory of Forest Ecology and Management, Institute of Applied Ecology, Shenyang, China; ^4^ North Central Agriculture Research Laboratory, USDA-ARS, Brookings, SD, United States

**Keywords:** rhizosphere processes, soil carbon cycle, stress environments, climate change, drying-rewetting

Root-soil-microbe interactions play a vital role in influencing soil nutrient mobilization and acquisition, which significantly impacts the soil carbon cycle (Lin et al., 2023). Over the last decade, extreme events, such as drought, flood, heat wave, and temperature fluctuations have occurred frequently. Flooding exerts an indirect effect on plant C:N:P stoichiometry primarily by bringing about modifications in soil factors and plant community composition (Wang et al.). It is necessary to reveal how plant-associated rhizosphere processes affect soil carbon loss under these stress environments. For example, soil stores a large amount of carbon, but many studies ignore the effect of plant roots on soil carbon dynamics. There is still a lack of research on the impact of rhizosphere processes on soil carbon balance under stress environments, which limits the accuracy of correctly predicting the feedback relationship between soil carbon pool and climate change. It is difficult to quantify and predict the terrestrial carbon cycle under climate change because of the limited understanding of the rhizosphere processes, which can mediate the transformation or recycling of carbon and related nutrients in terrestrial ecosystems, especially under stressful environments. The rhizosphere is conducive to the accumulation of soil carbon and nutrients, microbial growth and metabolism, as well as the formation of carbon degradation enzymes. Roots can produce rhizosphere effects to acquire available nitrogen, alleviate nitrogen limitation and sustain nitrogen nutrition by regulating nitrogen cycling in the rhizosphere (Duan et al.). Therefore, more carbon is lost through microbial respiration, leading to accelerated decomposition of carbon in the rhizosphere (Lv et al., 2023). Rhizosphere priming effect via living roots induced soil organic carbon mineralization by 70–204%, which was ascribed to the coupled biotic (microbial activation and microbial N-mining)-abiotic (iron- (Fe-) (hydr-) oxide) processes (Jiang et al., 2021). The rhizosphere priming effect will increase the soil organic carbon decomposition rate of northern circumpolar permafrost by 12%, and the absolute carbon loss of the soil will reach 40 Pg by 2100. The variations in temperature and precipitation can mediate structure and function of rhizosphere microbiomes, thus impacting soil carbon cycle and climate change (Song et al.). It is therefore suggested that the rhizosphere priming effect should be fully considered in the carbon model to accurately predict and control global warming (Keuper et al., 2020). In the previous studies on the decomposition of soil organic matter, soil incubation or field root-exclusion was typically used, thus ignoring the rhizosphere effect (Zhao et al., 2023). In future studies, more attention should be paid to the rhizosphere effect or/and the rhizosphere priming effect on SOM decomposition in response to environmental stress for better modelling. Besides, stressful environments exert impacts on root activities and belowground carbon input through influencing plant physiology, root exudation and rhizosphere microbial activities, thereby indirectly influencing the decomposition of soil organic matter in the rhizosphere and its responses to climate change (Xia et al., 2022). Nevertheless, research concentrating on environmental stress, particularly the effects of drought, flood, heat wave, and temperature fluctuations on rhizosphere processes and carbon balance, is extremely scarce. More investigations should be carried out to ascertain the influence of rhizosphere processes on the soil carbon cycle under stressful circumstances.

Extreme climate events refer to weather and climate phenomena with a historical recurrence rate of less than 10% or breaking historical extremes, and causing significant disastrous impacts. They are mainly characterized by low occurrence frequency, high event intensity, and significant social influence. In recent years, under the superimposed influence of multiple factors such as global warming, El Niño and La Niña phenomena, the occurrence frequency of extreme weather events has an increasing trend. Extreme climates have a significant impact on soil carbon pools. For instance, extreme drought reduces soil heterotrophic respiration during the plant growth recession period, as well as bacterial diversity and the abundance of related functional groups. Heavy rain increases surface runoff and accelerates the erosion of surface soil organic carbon. Extreme cold and drought deteriorate the living environment of organisms and reduce the input of organic matter. High temperatures and droughts decrease plant productivity, thereby reducing the input of organic carbon into the soil. Fungi are less prone to being affected by environmental changes compared to bacteria and have a significant role in enhancing soil organic matter within the rhizosphere (Wang et al.). Different types of extreme climates have varying effects. For example, coarse-textured soils suffer greater carbon losses under extreme climates. In the future, approximately 88% of regions will experience extreme events of negative net biome productivity with intensities greater than positive ones, accelerating the weakening of terrestrial carbon sinks. To address this, it is necessary to enhance monitoring and research, promote sustainable land management and agricultural practices, strengthen vegetation protection and restoration, and improve policies and regulations.

The above content suggests strengthening research on the key role of root-soil-microbe interactions in the soil carbon cycle, especially under extreme climate events. Current research deficiencies include ignoring the role of plant roots, limited research on the impact of rhizosphere processes on soil carbon balance, and insufficient understanding of rhizosphere processes, which complicates quantifying and predicting the terrestrial carbon cycle and understanding the feedback between carbon cycle and climate change. Some uncertainties exist in evaluating the terrestrial carbon cycle under increased environmental stress (**Figure 1**), including: (i) Rhizodeposition and influencing factors: How environmental stress affects plant production and the quantity and quality of rhizodeposits of plants determines the carbon input to the soil. (ii) Rhizosphere priming and regulatory factors: The rhizosphere priming effect is a key link in controlling carbon output in the rhizosphere, but how it responds to environmental stress still lacks in-depth research. (iii) Temperature sensitivity and Birch effect: The decomposition rate of soil organic matter, as well as rhizodeposition and rhizosphere priming effect, can be changed by warming and the drying-rewetting process of the soil. The extent of the change determines the feedback between climate change and soil carbon pool, but current understanding is very limited.

**Figure 1:**
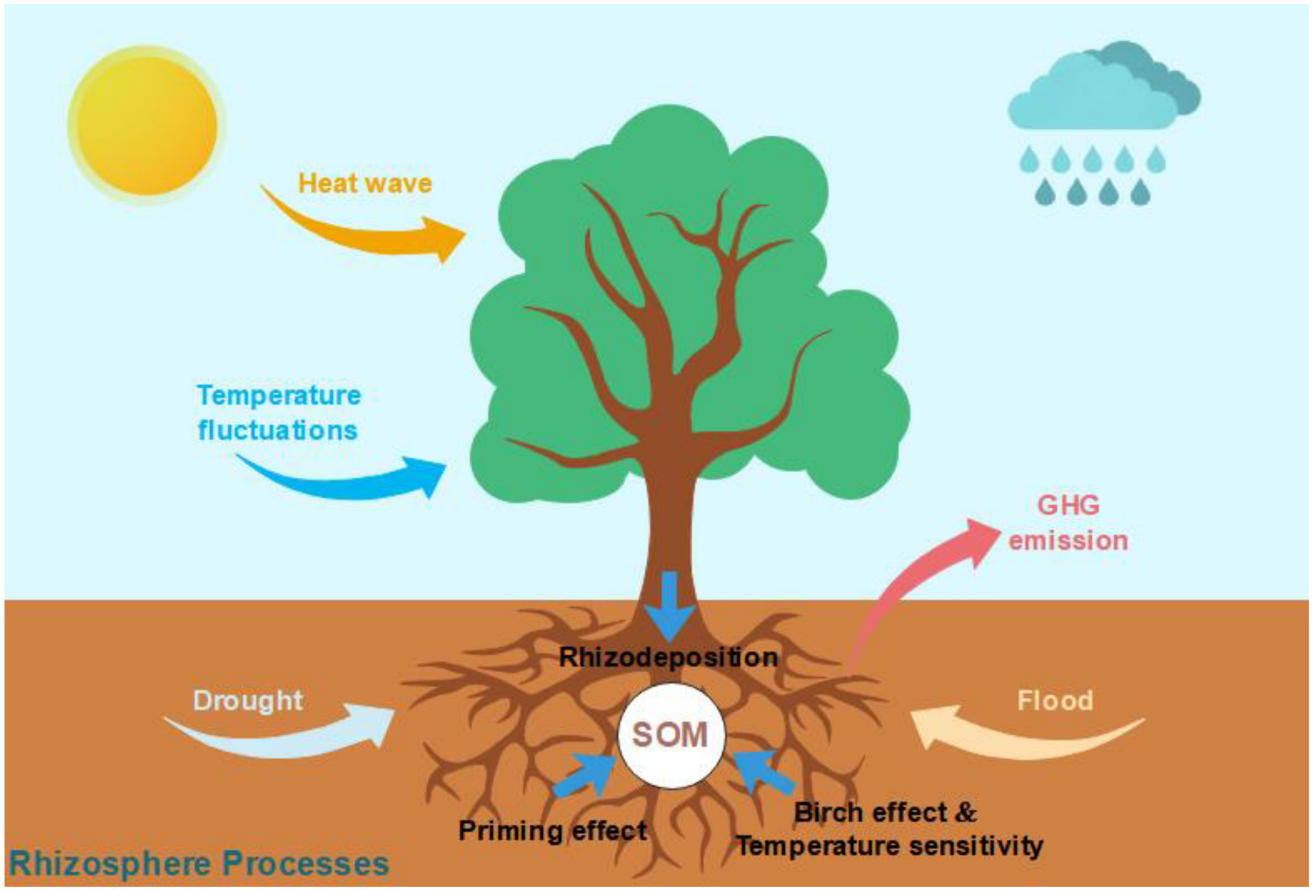
Schematic of stress environments on soil carbon cycle in rhizosphere processes.

The main goal of this Research Topic is to gain a deeper understanding of the terrestrial carbon cycle in relation to rhizosphere processes. This knowledge could contribute to strategies for mitigating the impacts of environmental stress on carbon cycling and developing sustainable land management practices. The research aims to reveal the drivers and mechanisms of rhizosphere processes on soil carbon formation, stabilization, and mineralization and emphasize the importance of these processes on ecosystem carbon source or sink function. It is anticipated that the research findings of this Research Topic will offer significant contributions to the understanding of rhizosphere processes on the soil carbon cycle under adverse circumstances for scholars and practitioners in related fields, including ecologists, soil scientists, and environmentalists. The guest editors highlight that intensified stress environments, such as drought, flood, heat wave, and temperature fluctuations, occur frequently, and are likely to bring about a substantial drop in soil C storage and an augmented contribution to the concentration of atmospheric CO_2_ by the rhizosphere processes. Consequently, greater emphasis needs to be placed on measures for carbon management that deal with the intensification of stress environments. Experiments on *in-situ* adult species are highly essential for optimizing ecological models and clarifying the influences of stress environments on the soil carbon cycle in the rhizosphere. Thus, an interdisciplinary approach integrating agricultural science, pedology, botany, environmental and ecological science is instantly demanded to confront the challenges associated with the rhizosphere carbon cycle in relation to stressful environments. By combining knowledge and information from various disciplines, a more comprehensive understanding of complex dynamics can be obtained, and effective strategies can be devised to alleviate the impacts of stress environments on the rhizosphere carbon cycle.

## Author contributions

JJL: Writing – original draft, Writing – review & editing, Conceptualization, Investigation. ZX: Writing – review & editing. PW: Writing – review & editing, Methodology. JYL: Writing – review & editing, Conceptualization. CY: Writing – review & editing, Conceptualization.

## References

[B1] Jiang Z. Liu Y. Yang J. Brookes P. C. Gunina A. (2021). Rhizosphere priming regulates soil organic carbon and nitrogen mineralization: The significance of abiotic mechanisms. Geoderma 385, 114877. doi: 10.1016/j.geoderma.2020.114877

[B2] Keuper F. Wild B. Kummu M. Beer C. Blume-Werry G. Fontaine S.. (2020). Carbon loss from northern circumpolar permafrost soils amplified by rhizosphere priming. Nat. Geosci. 13, 560–565. doi: 10.1038/s41561-020-0607-0

[B3] Lin J. Hui D. Kumar A. Yu Z. Huang Y. (2023). Editorial: Climate change and/or pollution on the carbon cycle in terrestrial ecosystems. Front. Environ. Sci. 11. doi: 10.3389/fenvs.2023.1253172

[B4] Lv C. Wang C. Cai A. Zhou Z. (2023). Global magnitude of rhizosphere effects on soil microbial communities and carbon cycling in natural terrestrial ecosystems. Sci. Total Environ. 856, 158961. doi: 10.1016/j.scitotenv.2022.158961 36155049

[B5] Xia Z. He Y. Zhu Z. Korpelainen H. Li C. (2022). Covariations and trade-offs of phosphorus (P) acquisition strategies in dioecious Populus euphratica as affected by soil water availability. Funct. Ecol. 36, 3188–3199. doi: 10.1111/1365-2435.14193

[B6] Zhao Y. Lin J. Cheng S. Wang K. Kumar A. Yu Z.-G.. (2023). Linking soil dissolved organic matter characteristics and the temperature sensitivity of soil organic carbon decomposition in the riparian zone of the Three Gorges Reservoir. Ecol. Indic. 154, 110768. doi: 10.1016/j.ecolind.2023.110768

